# Multijoint Continuous Motion Estimation for Human Lower Limb Based on Surface Electromyography

**DOI:** 10.3390/s25030719

**Published:** 2025-01-24

**Authors:** Yonglin Han, Qing Tao, Xiaodong Zhang

**Affiliations:** 1 School of Mechanical Engineering, Xinjiang University, Urumqi 830047, China; 2 School of Mechanical Engineering, Xi’an Jiaotong University, Xi’an 710049, China

**Keywords:** sEMG, different movement patterns, TCN, multijoint angle estimation, TCN, CBAM

## Abstract

The estimation of multijoint angles is of great significance in the fields of lower limb rehabilitation, motion control, and exoskeleton robotics. Accurate joint angle estimation helps assess joint function, assist in rehabilitation training, and optimize robotic control strategies. However, estimating multijoint angles in different movement patterns, such as walking, obstacle crossing, squatting, and knee flexion–extension, using surface electromyography (sEMG) signals remains a challenge. In this study, a model is proposed for the continuous motion estimation of multijoint angles in the lower limb (CB-TCN: temporal convolutional network + convolutional block attention module + temporal convolutional network). The model integrates temporal convolutional networks (TCNs) with convolutional block attention modules (CBAMs) to enhance feature extraction and improve prediction accuracy. The model effectively captures temporal features in lower limb movements, while enhancing attention to key features through the attention mechanism of CBAM. To enhance the model’s generalization ability, this study adopts a sliding window data augmentation method to expand the training samples and improve the model’s adaptability to different movement patterns. Through experimental validation on 8 subjects across four typical lower limb movements, walking, obstacle crossing, squatting, and knee flexion–extension, the results show that the CB-TCN model outperforms traditional models in terms of accuracy and robustness. Specifically, the model achieved R^2^ values of up to 0.9718, RMSE as low as 1.2648°, and NRMSE values as low as 0.05234 for knee angle prediction during walking. These findings indicate that the model combining TCN and CBAM has significant advantages in predicting lower limb joint angles. The proposed approach shows great promise for enhancing lower limb rehabilitation and motion analysis.

## 1. Introduction

Surface electromyography (sEMG), a bioelectrical signal reflecting muscle activity, has significant application value in lower limb motion estimation [1,2]. It can capture dynamic muscle activity information, which is strongly correlated with joint movements, providing key technological support for lower limb rehabilitation [3], motion control [4], and exoskeleton robotics [5]. Daily activities such as walking, stair climbing, sitting, and standing hold significant importance for research [6]. The continuous motion estimation of these movement patterns not only helps understand the complexity of human motion control but also provides a theoretical foundation and technical support for robot design and human–robot interaction system development [7]. However, maintaining accuracy in lower limb multijoint continuous motion estimation based on surface electromyography (sEMG) signals, under varying movement patterns and individual differences, has always been a long-standing challenge.

Currently, the research on lower limb motion intention recognition based on surface electromyography (sEMG) signals is mainly divided into two parts. One approach focuses on using sEMG signals to recognize discrete human movement patterns [8,9,10]. Many researchers focus on recognizing a wider range of movement patterns and achieving higher recognition accuracy using sEMG signals. The emphasis of these studies lies in feature extraction methods and classification algorithms. The other approach involves using sEMG signals to estimate the continuous motion of human joints [11,12,13]. Motion pattern classification based on sEMG signals can only predict predefined movement patterns and recognize a limited number of discrete postures. It cannot be directly used as input for a controller to achieve continuous control, which severely impacts the coordination and compliance of exoskeletons. As a result, researchers have gradually shifted the focus of theoretical and applied studies towards continuous motion estimation based on sEMG signals. In constructing regression models between sEMG signals and continuous lower limb joint motion, most researchers have focused on sEMG feature engineering and model selection to achieve better estimation performance. For example, L. F. Sommer et al. [14] proposed a Wiener model approach to estimate elbow joint angles using sEMG signals from arm muscles. Xie et al. [15] used a generalized regression neural network (GRNN) model optimized with the golden section algorithm (GS-GRNN), taking the root mean square features of sEMG signals from the thigh muscles and hip joint angles as inputs to predict lower limb joint angles. Liu et al. [16] proposed an improved feature-based convolutional neural network (CNN) model for the continuous estimation of knee joint angles. EMG signals and joint angles during normal walking were recorded using a 6-channel sEMG acquisition system and an optical motion capture system. The results show that the improved CNN model can predict knee joint angles with higher accuracy.

In recent years, many researchers have used deep learning methods to estimate continuous joint angles of human motion based on sEMG signals [17,18,19]. The temporal convolutional network (TCN) is a neural network architecture suitable for processing sequential data. Compared to recurrent neural networks (RNNs), TCNs have significant advantages in handling long-term dependencies and parallel computation [20]. For example, Chen et al. [21] proposed a large-scale temporal convolutional network (LS-TCN), focusing on continuous motion estimation based on sEMG signals. The model was used to predict the angles of 10 joints during six types of grasping movements, achieving an accuracy of 71.6%. Wang et al. [22] proposed a subject-independent TCN-BiLSTM deep learning model. Compared to the long short-term memory (LSTM), artificial neural network (ANN), and gated recurrent unit (GRU), the temporal convolutional network with bidirectional long short-term memory (TCN-BiLSTM) model demonstrated higher accuracy in estimating the angles and torques of the hip, knee, and ankle joints, proving its robustness and practicality. Du et al. [23] proposed a joint angle prediction model based on sEMG signals for the continuous estimation of wrist motion angle changes. The model combines the advantages of TCN and LSTM networks; TCN is used to extract local features and deep information from the sEMG signals, while LSTM leverages its temporal memory capability to compensate for the limitations of TCN in capturing long-term dependencies, thus improving prediction performance.

The main contributions of this study are as follows:

(1) A CB-TCN network is proposed, which combines TCN and CBAM for estimating the angles of the hip, knee, and ankle joints in the lower limb. TCN captures the temporal features, while CBAM enhances the model’s focus on critical moments and features through its attention mechanism, thereby improving prediction accuracy.

(2) The model’s performance is validated through four common lower limb movements, including gait, obstacle crossing, squatting, and knee flexion–extension. Its performance is tested under different movement scenarios and compared with the following four classic models: encoder–decoder temporal convolutional network (ED-TCN), TCN, LSTM, and Wiener filter. In this study, its advantages and performance improvements are comprehensively evaluated.

In this study, the aim is to evaluate the CB-TCN model for estimating joint angles during various daily activities using the envelope extracted from sEMG signals. The structure of this paper is organized as follows: Section 2 presents the experimental setup; Section 3 provides a detailed discussion of the research methods; Section 4 discusses the results and their analysis; and Section 5 concludes with the findings and outlines potential directions for future research.

## 2. Experimental Setup

### 2.1. Experimental Design

This study recruited 8 healthy volunteers (6 males and 2 females) with an average age of 23 ± 1.73 years, an average height of 173.75 ± 7.3 cm, and an average weight of 62.5 ± 11.46 kg. All participants signed informed consent forms and agreed to the use of experimental data and images for scientific research purposes. The experiment was approved by the Ethics Committee of Xi’an Jiaotong University, approval number: 2024YS239. All experimental procedures were conducted in accordance with the relevant provisions of the Declaration of Helsinki.

The experimental design included the following four types of movement patterns: gait, obstacle crossing, squatting, and knee flexion–extension. The specific arrangements were as follows:

1. Gait: Each participant completed this experiment in approximately 5.5 s, with a walking speed controlled at 2 m/s, performing a total of 5 trials. Gait involves the dynamic changes of lower limb joints during walking, which helps analyze the joint angle variations and gait cycle of normal walking. Although this speed (equivalent to 7.2 km/h) exceeds typical walking speeds (usually around 1.2–1.5 m/s), it was deliberately chosen to simulate brisk walking or rapid movement conditions, offering a more thorough evaluation of the model’s performance in such scenarios.
2. Obstacle Crossing: Each participant completed this experiment in approximately 5 s, performing a total of 5 trials. Obstacle crossing simulates lower limb movements when stepping over an obstacle, providing angle change data for rapid movements within a short time.
3. Squatting: Each participant completed this experiment in approximately 25 s, with 10 repetitions of each squatting action, performing a total of 5 trials. This movement pattern is designed to simulate the squatting and standing actions in daily life and is suitable for studying dynamic control and joint angle variations in the lower limbs.
4. Knee Flexion–Extension: Each participant completed this experiment in approximately 20 s, with 10 repetitions of each knee flexion–extension action, performing a total of 5 trials. This movement pattern primarily focuses on the flexion–extension of the knee joint and effectively tests the continuous variation in the knee joint angle.

During this experiment, participants were instructed to perform each task at their natural pace. A 5 min rest period was provided after each session to ensure recovery.

### 2.2. Data Collection

The continuous walking capability of the human lower limb results from the coordinated action of multiple muscles that drive the lower limb to perform the required movements. Each side of the lower limb consists of at least 30 muscles, working together to enable the movement across 7 degrees of freedom.

**Figure 1:**
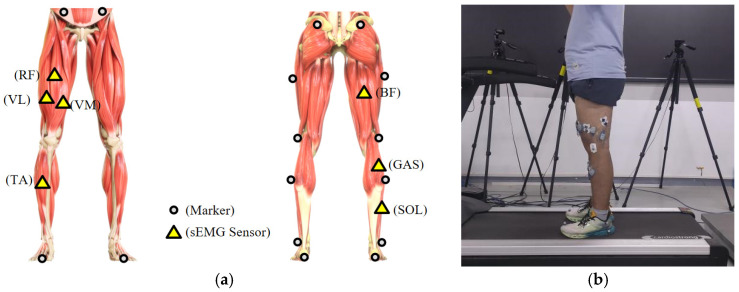
This shows the specific placement of the sensors, where (**a**) represents the arrangement of surface electromyography (sEMG) sensors and reflective markers on the right leg and (**b**) depicts the scenario of the participant performing the walking experiment on a treadmill.

To better collect surface electromyography (sEMG) signals closely related to lower limb movements, in this study, 7 major muscles were selected as targets for signal acquisition, including rectus femoris (RF), tibialis anterior (TA), soleus (SOL), gastrocnemius (GAS), vastus medialis (VM), vastus lateralis (VL), and biceps femoris (BF) [24]. The placement of the electromyography sensors followed the Surface ElectroMyoGraphy for the Non-Invasive Assessment of Muscles (SENIAM) guidelines to ensure optimal signal quality [25]. SENIAM provides standardized protocols for electrode placement to ensure consistent and reliable measurements of muscle activity. Additionally, the specific locations of reflective markers and sEMG sensor placements during lower limb movements are shown in Figure 1.

In this study, a VICON motion capture system consisting of 10 cameras was employed, primarily used to collect motion data from 16 motion capture markers placed on the lower limbs. The sampling frequency was set to 100 Hz. In this experiment, a total of seven surface electromyography sensors were used to record electromyographic signals, utilizing Noraxon wireless equipment with a sampling frequency of 2000 Hz. In this experiment, the MX-Giganet module was used to connect the host PC and MX cameras, serving as an interface between the VICON system and the sEMG system, ensuring synchronized data acquisition, recording, and analysis of both systems in the Vicon Nexus 2.7. This configuration maximized signal synchronization and minimized signal deviation caused by hardware and software errors. In this study, the placement of the cameras and markers followed standard motion capture protocols and complied with the operational specifications of the VICON system, ensuring the accuracy of data collection.

### 2.3. Data Processing

(1) Preprocessing of sEMG Signals: In this study, a fourth-order Butterworth filter (20–450 Hz) was applied to preprocess the electromyographic signals, removing both high-frequency and low-frequency noise. Additionally, to eliminate the influence of power line interference on the sEMG signal quality, a 50 Hz notch filter (Q factor = 35) was used to filter out power line noise. Subsequently, a 10 Hz low-pass filter was applied to extract the envelope of the electromyographic signals.

(2) Preprocessing of Motion Signals: The motion data, acquired from a high-precision motion capture system, were preprocessed using a fourth-order zero-phase Butterworth low-pass filter (cutoff frequency of 15 Hz) and a critically damped low-pass filter (cutoff frequency of 15 Hz) to remove high-frequency noise and smooth the signals [26]. The joint angles were then derived using the cosine formula, based on the motion capture data, ensuring accurate estimation of the joint angles from the positions of the relevant body segments.

In addition, to address the issue of inconsistent sampling frequencies, resampling of the preprocessed sEMG signals was performed. The sampling frequency of the joint angle signals was 100 Hz, while that of the sEMG signals was 2000 Hz. Since the bandwidth of lower limb joint movements is relatively low, the requirements for the sampling frequency are less stringent. To reduce computational load, all signals were resampled to a unified sampling frequency of 20 Hz.

### 2.4. Sliding Window Data Expansion Strategy

In this study, a sliding window data expansion strategy was employed, dividing the original time-series data into overlapping segments, each serving as an independent training sample [27,28]. The window size (W) was 10, with a step size (S) of 1, allowing each time point to be the center of multiple windows. This increases the number of training samples and helps the model capture fine-grained temporal patterns, enhancing prediction accuracy and generalization. The core idea of the above sliding window data expansion strategy can be expressed by the following formula:

Assume the original time-series data are represented as $X=\left[x_{1},x_{2},\cdots ,x_{T}\right]$, where $x_{t}$ represents the data at the t-th time step, where *T* is the total length of the data. It is defined the size of the sliding window as W (in this case, 10), and the step size as S (in this case, 1). The window $w_{i}$ represents a continuous set of *W* data points starting from the *i*-th time step, specifically, as follows:

(1) $$w_{i}=\left[x_{i},x_{i+1},\cdots ,x_{i+w-1}\right]$$

For i=1,2,⋯,T−w+1, each window contains *w* consecutive data points. By setting the step size *S* = 1, the window slides one-time step at a time, generating multiple overlapping windows.

Therefore, the set of windows is as follows:

(2) $$W=\left\{w_{1},w_{2},\cdots w_{T-W+1}\right\}$$

Each window is used as an independent input to the model, allowing it to capture temporal dependencies and local features in the data.

## 3. Methods

### 3.1. TCN

TCN is a deep learning model based on convolution operations, widely used for processing time-series data [29]. Compared to traditional RNNs, TCN provides an efficient alternative, excelling in time-series modelling tasks due to its parallel computation and longer memory capability. Its main structure includes causal convolutions, dilated convolutions, and residual blocks [30].

#### 3.1.1. Causal Convolution

**Figure 2:**
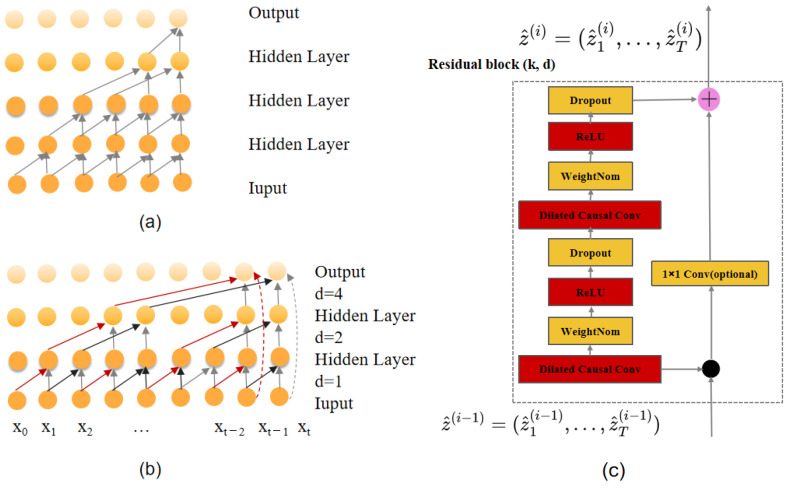
The structure of the temporal convolutional network (TCN), including (**a**) causal convolution, (**b**) dilated convolution, and (**c**) residual module.

The core goal of causal convolution is to ensure that the model’s output only depends on the current and past input data, and not on future inputs, thus strictly adhering to the principle of causality, as shown in Figure 2a.

Given that the convolution filter is represented as $F=(f_{1},f_{2},\cdots ,f_{k})$ and the input sequence as $X=(x_{1},x_{2},\cdots x_{T})$, the causal convolution at position t can be represented as follows:

(3) $$y_{t}^{causal}=\sum_{k=1}^{K}f_{k}x_{t-k}$$

Here, *k* is explicitly defined as the kernel size, which determines the number of coefficients in the convolution filter.

#### 3.1.2. Dilated Convolution

Dilated convolution can achieve a larger receptive field through interval sampling, compensating for the limitations of causal convolution, as shown in Figure 2b.

Given that the convolution filter is represented as $F=(f_{1},f_{2},\cdots ,f_{k})$ and the input sequence is denoted as $X=(x_{1},x_{2},\cdots x_{T})$, the dilated convolution at position t can be represented as follows:

(4) $$y_{t}^{\mathrm{dilated}}=\sum_{k=1}^{K}f_{k}x_{t-d\cdot k}$$

Here, *k* is the number of coefficients in the convolution filter. *d* is the dilation rate, controlling the spacing between the elements of the input sequence used in the convolution.

#### 3.1.3. Residual Module

TCN uses residual modules to mitigate the problems of vanishing and exploding gradients that occur as the network deepens, allowing information to be transmitted stably across layers and thereby improving generalization ability, as shown in Figure 2c. The residual module in TCN includes causal convolution, batch normalization (BN) layers, and ReLU activation functions. In the residual module, a dropout layer is also added for regularization to prevent overfitting. The input of the residual module is added to the output after two convolution operations through an identity mapping (or a 1 × 1 convolution) to ensure that the dimensions are consistent.

### 3.2. CBAM

CBAM is an integrated module that incorporates both spatial attention and channel attention mechanisms. By using the CBAM attention mechanism, it focuses on important features while suppressing irrelevant ones [31].

**Figure 3:**
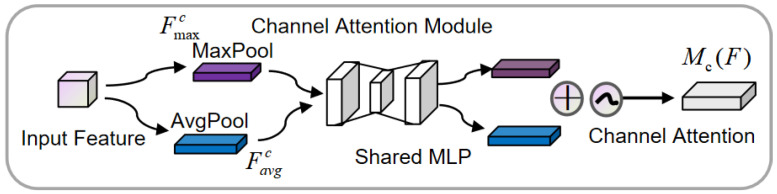
Channel attention module.

Channel attention focuses on the most meaningful features by increasing the weights of effective channels and reducing the weights of less effective ones, as shown in Figure 3. The input feature map is first processed by global max pooling and global average pooling based on width and height, respectively. Then, the outputs of the pooling operations are passed through an MLP. The features output by the MLP are then summed element-wise, followed by a sigmoid activation to generate the final channel attention feature map. This channel attention feature map is then element-wise multiplied with the input feature map to generate the input features required for the spatial attention module.

(5) $$\begin{array}{c} F_{avg}^{c}=AvgPool(F)\in R^{C},F_{\max}^{c}=MaxPool(F)\in R^{C}\\ M_{c}(F)=\sigma (W_{1}(R\mathrm{eLU}(W_{0}(F_{\mathrm{avg}}^{c})))+W_{1}(R\mathrm{eLU}(W_{0}(F_{\max}^{c})))) \end{array}$$

Here, σ is the sigmoid activation function; $W_{0}\in R^{C\times k}$ and $W_{1}\in R^{k\times 1}$ represent the weights; and k is the number of attention units. $F\in R^{C\times H\times W}$ denotes the feature map, and the channel attention weights $M_{c}(F)\in R^{C}$ are within the range [0, 1], where C is the number of channels, and H and W represent the height and width of the feature map.

**Figure 4:**
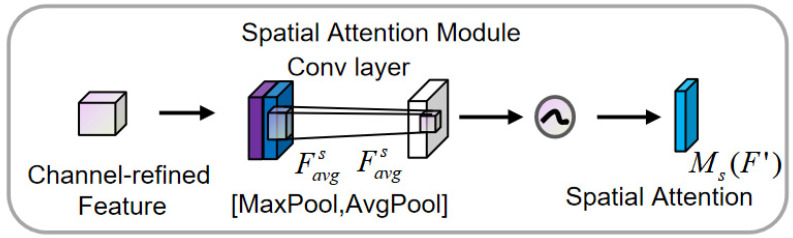
Spatial attention module.

Spatial attention obtains two different features by using max pooling and average pooling along the channel dimension, as shown in Figure 4. The feature map output by the channel attention module is used as the input feature map for this module. First, a global max pooling and global average pooling are performed based on channels. The results of these two operations are then concatenated along the channel dimension. After that, a convolution operation is applied to reduce the dimensionality to a single channel. The resulting feature map is passed through a sigmoid function to generate the spatial attention feature. Finally, this feature is element-wise multiplied with the input feature map of the module to obtain the final generated feature.

$$F_{avg}^{s}=AvgPool(F^{\prime })\in R^{C},F_{\max}^{s}=MaxPool(F^{\prime })\in R^{C}$$

(6) $$F_{\mathrm{cat}}^{s}=[F_{avg}^{s};F_{\max}^{s}],F_{\mathrm{cat}}^{s}\in R^{2\times T}$$

(7) $$M_{s}(F^{\prime })=\sigma (Conv1D(F_{\mathrm{cat}}^{s}))$$

In the equation, σ is the sigmoid activation function; the size of the convolution kernel is typically 3; and $M_{s}(F^{\prime })\in R^{T}$ represents the spatial attention weight.

### 3.3. CB-TCN

The CB-TCN proposed in this paper is a deep learning model that combines the temporal convolutional networks (TCNs) and convolutional block attention module (CBAM). The structure is CB-TCN (temporal convolutional network + convolutional block attention module + temporal convolutional network). CBAM enhances the feature extraction capability, while the two TCN layers at the front and back efficiently model sequential data. This architecture is suitable for tasks such as continuous motion regression and other time-series prediction tasks.

**Figure 5:**
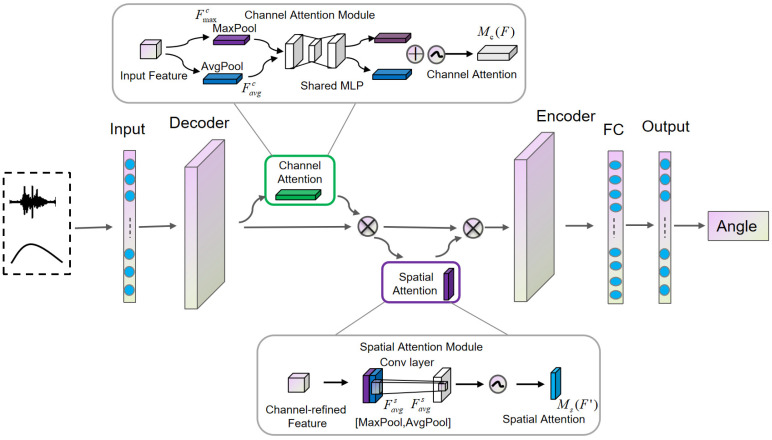
The complete framework of the proposed CB-TCN model for angle estimation.

The overall framework of the CB-TCN model is shown in Figure 5. The model adopts a cascaded structure of TCN → CBAM → TCN, where the initial TCN module extracts temporal features from the input time-series data. Then, the convolutional block attention module (CBAM) enhances the extracted features by emphasizing the most relevant temporal and spatial information. Finally, the second TCN module further models the enhanced features and outputs the predicted joint angles. In this architecture, the initial TCN module can be seen as the encoder, responsible for extracting temporal features from the original input sequence and transforming them into latent feature representations, while the second TCN module acts as the decoder, modeling the enhanced features and generating the final output prediction. This design fully leverages the advantages of TCN in temporal feature extraction and CBAM in feature refinement, ensuring accuracy and robustness when modeling complex temporal dependencies.

In this study, 500 iterations were set to observe the trend of the model’s loss curve. After extensive experimental testing, 50 training epochs were selected, which fully met the loss requirements. The mean squared error (MSE) loss function was used as the model’s loss function, and L2 regularization (weight_decay) along with the Adam optimizer were employed during the training process. MSE loss is used as the loss function to measure the difference between the predicted and actual values. MSE loss is calculated by averaging the squared differences between the predicted values and the actual ground truth values. L2 regularization helps prevent overfitting by penalizing large weight values, while the Adam optimizer combines the advantages of momentum and adaptive learning rates, enabling more efficient parameter updates, faster training, and stable convergence [32]. This approach also incorporates a learning rate scheduler, with the initial learning rate set to LR = 0.001, and the learning rate decayed to 50% of its original value every 10 epochs (i.e., gamma = 0.5) [33]. A batch size of 50 was used throughout the training process.

### 3.4. Evaluation Metrics

In this paper, the root mean square error (RMSE), normalized root mean square error (NRMSE), and coefficient of determination (R^2^) were introduced to evaluate the model’s estimation results. RMSE measures the average error between the predicted and actual values, with a lower RMSE indicating more accurate predictions. NRMSE normalizes RMSE according to the range of observed values, making it easier to compare errors across different datasets. R^2^ represents the model’s ability to explain the variance in the observed data, with values close to 1 indicating a good fit and values close to 0 indicating poor prediction performance. The formulas for RMSE, NRMSE, and R^2^ are as follows:

(8) $$R^{2}=1-\frac{\sum_{t=1}^{N}{\left({\hat{y}}_{t}-y_{t}\right)}^{2}}{\sum_{t=1}^{N}{\left(\bar{y}-y_{t}\right)}^{2}}$$

(9) $$\mathrm{RMSE}=\sqrt{\frac{1}{N}{\sum_{t=1}^{N}\left({\hat{y}}_{t}-y_{t}\right)}^{2}}$$

(10) $$\mathrm{NRMSE}=\frac{\mathrm{RMSE}}{\max(y_{t})-\min(y_{t})}$$

where ${\hat{y}}_{t}$ is the predicted value at time *t*; $y_{t}$ is the measured value at time *t*; $\bar{y}$ is the mean of the observed values; and *N* is the number of samples.

### 3.5. Model Comparison

(1) ED-TCN

ED-TCN is an enhanced version of the traditional TCN that uses an encoder–decoder architecture to more effectively model sequential data, particularly for time-series prediction tasks. In this architecture, ED-TCN employs two separate TCN layers: one serves as the encoder to extract temporal features from the input, while the other acts as the decoder to transform these features into the final prediction results. This model shares the same hyperparameter settings as CB-TCN, including the kernel size, the number of convolution layers, and other parameters.

(2) TCN

TCN is a CNN-based model specifically designed to handle time-series data. By using causal convolutions and dilated convolutions, it effectively captures dependencies over long time horizons while avoiding an increase in computational load. TCN shares the same hyperparameter settings as CB-TCN, such as the kernel size, the number of convolution layers, and other parameters.

(3) LSTM

LSTM is a special type of recurrent neural network (RNN) that effectively captures long-term dependencies in sequences. Through gating mechanisms such as forget gates, input gates, and output gates, LSTM is able to retain important information in time series while ignoring irrelevant information, thereby improving the performance and stability when handling long sequences. This model shares the same hyperparameter settings as CB-TCN, including the kernel size, the number of layers, and other parameters.

(4) Wiener Filter

The Wiener filter is a classical linear filter widely used in signal processing and noise suppression. Its goal is to optimize the difference between the input signal and the output signal by minimizing the mean squared error (MSE). In time-series prediction, the Wiener filter is commonly used for denoising and smoothing signals, but due to its linear nature, it is generally not suitable for capturing complex nonlinear dependencies.

## 4. Results

In this study, sEMG envelope signals and joint angles are used from four different motion modes (walking, obstacle crossing, squat, and knee flexion–extension) as inputs to the model. The evaluation metrics include R^2^, RMSE, and NRMSE. All metric values are obtained through 5-fold cross-validation and used as the final results. In the flexion–extension movement, the focus is primarily on the actions of the knee and ankle joints, as the range of motion and variations in the hip joint are relatively limited. Therefore, the hip joint is typically not analyzed in detail.

### 4.1. Angle Estimation Curve Analysis

**Figure 6:**
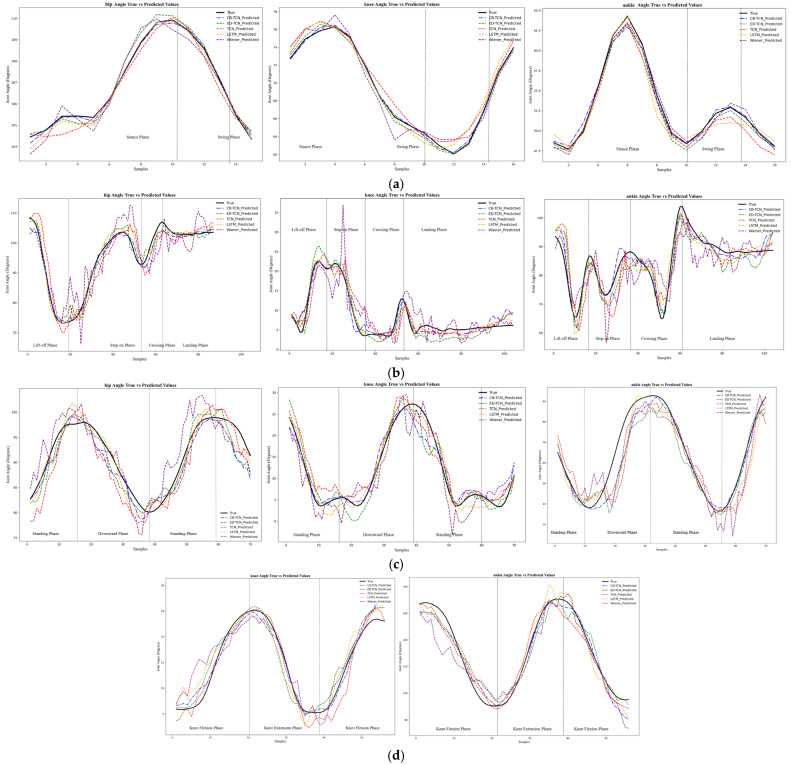
(**a**) Gait motion; (**b**) obstacle crossing motion; (**c**) squat motion; (**d**) knee flexion–extension motion.

Figure 6 shows the estimation curves for the hip, knee, and ankle joints of the lower limb using five different models. The black solid line represents the actual joint curve, the blue dashed line represents the predictions of the CB-TCN model, the green dashed line represents the predictions of the ED-TCN model, the red dashed line represents the predictions of the TCN model, the yellow dashed line represents the predictions of the LSTM model, and the purple dashed line represents the predictions of the Wiener model. A comparison of the model structure parameters is provided in Table S1.

As shown in Figure 6, all models effectively capture the variations in joint angles. However, there are noticeable differences between the models. The proposed CB-TCN model demonstrates superior performance, with its predicted curve closely following the actual data, showing better fitting accuracy compared to the other models. The following is an analysis of the prediction curves for different motions:

(1) Gait Motion

In gait motion, the joint angles exhibit a periodic pattern due to the repetitive gait cycle, fluctuating in a regular manner. All models are able to capture these periodic patterns, but there are subtle differences in their accuracy in tracking the actual joint angle changes. Notably, the CB-TCN model aligns more precisely with the actual data, especially during transitional phases (e.g., from the stance phase to the swing phase), highlighting its exceptional ability to model the dynamic changes in the gait cycle. The subtle differences observed in the other models suggest that they may struggle to capture the fine-grained motion details that are crucial for walking.

(2) Obstacle Crossing Motion

In the obstacle crossing motion, the joint angles undergo sudden changes due to the dynamic shift in body posture during the stepping phase. While all models are able to capture the overall trend, the CB-TCN model excels in handling these sudden changes, closely following the actual data, particularly when the foot crosses the obstacle. In contrast, other models exhibit more noticeable jitter at these moments, indicating a lower prediction capability for such rapid posture adjustments.

(3) Squat and Stand Motion

As the body lowers and rises, the squat and stand motion causes significant changes in joint angles, especially in the knee and hip joints. The prediction curves of all models generally align well with the actual data, but the CB-TCN model stands out as it more smoothly and accurately depicts the rising and lowering movements, particularly the knee joint angle. The subtle differences observed in the other models may suggest difficulties in adapting to the complex nonlinear dynamic characteristics of the squat and stand motion.

(4) Knee Flexion–Extension Motion

The knee flexion–extension motion exhibits relatively simple but rapid fluctuations, especially during the knee flexion and extension phases. Due to the sharp transitions in this motion, accurately predicting these changes can be more challenging. During this experiment, as the subject was seated on a chair, the knee and ankle joints exhibited significant changes during knee flexion–extension, while the hip joint showed minimal changes, and the captured curve changes were irregular. Therefore, the model’s estimation of the hip joint motion during knee flexion–extension is not presented here. Similarly, the CB-TCN model performs better, closely following the actual data and accurately capturing the subtle variations in the knee joint angle. Other models tend to show some lag or less smooth transitions in their prediction curves, suggesting that their ability to capture rapid and fine motions may be limited.

The CB-TCN model consistently demonstrates outstanding performance in tracking joint angle changes across various motions, particularly in motion transitions and certain high-dynamic scenarios. It excels at capturing fine motion details and rapid transitions, which are crucial for understanding joint behavior in activities such as walking, obstacle crossing, squatting, and knee flexion–extension. However, the CB-TCN model has some limitations when handling fast and complex movements, such as squatting and obstacle crossing. Specifically, the model performs poorly in capturing rapid changes in joint angles, particularly in situations with fast dynamic changes, making it difficult to accurately predict joint motion trajectories. Additionally, muscle activation patterns are typically nonlinear, and the model has not effectively handled this complex, time-varying muscle response, leading to larger prediction errors in certain complex movements. To improve the model’s accuracy and robustness, multi-task learning or multi-scale feature learning methods can be introduced to better capture the interactions and coordination between joints, enhancing the model’s ability to model multi-joint coordination in lower limb movements. Furthermore, increasing training data with more complex movement patterns can enhance the model’s generalization ability, ensuring that it maintains good predictive accuracy across different movement scenarios and practical applications.

### 4.2. Comparison Analysis of the CB-TCN Model with Other Models

**Figure 7:**
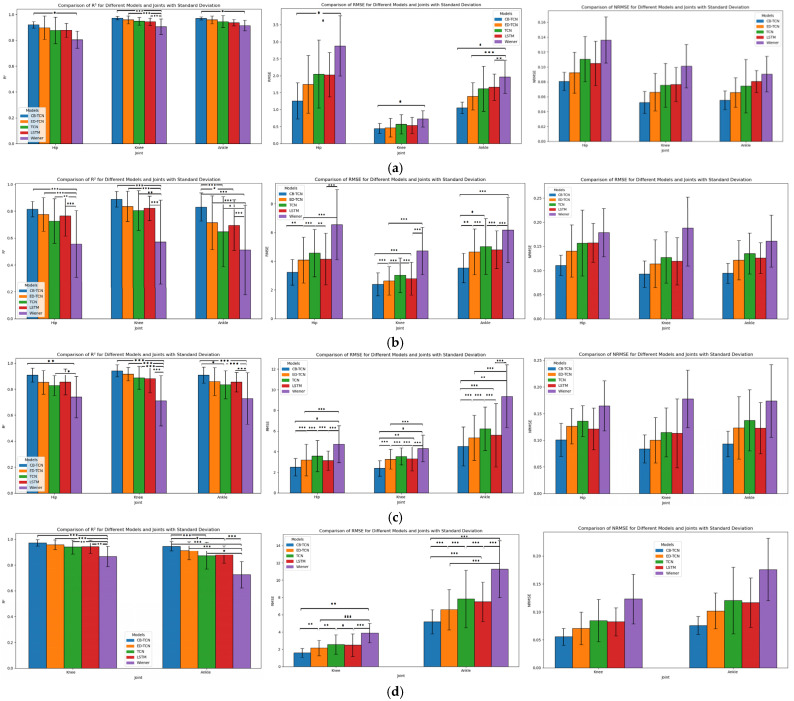
The R^2^, RMSE, and NRMSE of five models across four movements (hip, knee, ankle) show statistically significant differences in R^2^ and RMSE (*p* < 0.05; *: 0.05 ≥ *p* > 0.001; **: 0.001 ≥ *p* > 0.0001; ***: *p* < 0.0001), with the results averaged over 8 subjects. (**a**) Gait motion; (**b**) obstacle-crossing motion; (**c**) squat motion; (**d**) knee flexion–extension motion.

In the comparison of the five models (CB-TCN, ED-TCN, TCN, LSTM, Wiener) in terms of R^2^, the RMSE was performed using a one-way analysis of variance (ANOVA), followed by a post hoc Tukey test with a significance level of 0.05. As shown in Figure 7, there are statistically significant differences (*p* < 0.05) in the R^2^ and the RMSE values for the hip, knee, and ankle joint motions among the five models. Figure 7 shows the mean values of R^2^, RMSE, and NRMS for the eight participants. Table S2 summarizes the average statistical metrics for all participants.

(1) Gait Motion

Hip Joint: The R^2^ of the CB-TCN model is 0.9190, which is the best among all models. It outperforms the second-best model, ED-TCN (R^2^ = 0.8965), by a margin of 0.0225 and shows a more significant improvement of 0.1148 compared to the Wiener model. The RMSE is 1.2648°, also lower than the other models, particularly the Wiener model (a difference of 1.6318°). For NRMSE, CB-TCN has a value of 0.08075, which is lower than the other models.

Knee Joint: The R^2^ of the CB-TCN model is 0.9718, significantly higher than the other models. It outperforms the second-best model, ED-TCN (R^2^ = 0.9579), by 0.0139 and shows a notable improvement of 0.0667 compared to the Wiener model. In terms of RMSE, CB-TCN is 0.4991°, lower than the other models, particularly the Wiener model (a difference of 0.2234°). For NRMSE, CB-TCN has a value of 0.05234, which is lower than the other models.

Ankle Joint: The R^2^ of the CB-TCN model is 0.9701, which outperforms the other models. It shows an improvement of 0.0121 compared to the second-best model, ED-TCN (R^2^ = 0.9580), and a notable difference of 0.0566 compared to the Wiener model. The RMSE is 1.0526°, outperforming the other models, particularly the Wiener model (a difference of 0.9147°). For NRMSE, CB-TCN has a value of 0.05531, which is also lower than the other models.

(2) Obstacle Crossing Motion

Hip Joint: The R^2^ of the CB-TCN model is 0.8220, which is superior to the other models. It demonstrates an improvement of 0.0432 compared to the second-best model, ED-TCN (R^2^ = 0.7788), and a significant difference of 0.2954 compared to the Wiener model. In terms of RMSE, CB-TCN is 3.2414°, lower than the other models, especially the Wiener model (a difference of 3.3052°). For NRMSE, CB-TCN has a value of 0.112, which is lower than the values of the other models.

Knee Joint: The R^2^ of the CB-TCN model is 0.8999, which outperforms the other models. It shows an improvement of 0.0485 compared to the second-best model, ED-TCN (R^2^ = 0.8514), and a substantial difference of 0.2879 compared to the Wiener model. The RMSE is 2.4017°, lower than the other models, especially the Wiener model (a difference of 2.3222°). For NRMSE, CB-TCN has a value of 0.09267, which is lower than the other models.

Ankle Joint: The R^2^ of the CB-TCN model is 0.8315, which is higher than the other models. It shows an improvement of 0.11685 compared to the second-best model, ED-TCN (R^2^ = 0.71465), and a substantial difference of 0.3200 compared to the Wiener model. The RMSE is 3.5349°, relatively low, especially compared to the Wiener model (a difference of 1.2344°). For NRMSE, CB-TCN has a value of 0.0945, which is comparatively lower than the values of the other models.

(3) Squat and Stand Motion

Hip Joint: The R^2^ of the CB-TCN model is 0.9089, which outperforms the other models. It shows an improvement of 0.05605 compared to the second-best model, ED-TCN (R^2^ = 0.85285), and a significant difference of 0.1689 compared to the Wiener model. The RMSE is 2.4053°, lower than the other models, particularly the Wiener model (a difference of 1.9422°). For NRMSE, CB-TCN has a value of 0.1008, which is lower than the values of the other models.

Knee Joint: The R^2^ of the CB-TCN model is 0.9406, which is significantly higher than the other models. It shows an improvement of 0.02535 compared to the second-best model, ED-TCN (R^2^ = 0.91525), and a notable difference of 0.2308 compared to the Wiener model. The RMSE is 2.5121°, lower than the other models, particularly the Wiener model (a difference of 2.2364°). For NRMSE, CB-TCN has a value of 0.08422, which is lower than the other models.

Ankle Joint: The R^2^ of the CB-TCN model is 0.9092, which demonstrates a clear advantage over the other models, particularly the Wiener model, with a difference of 0.1803. Compared to the second-best model, ED-TCN (R^2^ = 0.8587), CB-TCN also shows a notable improvement. The RMSE is 4.7484°, relatively low, especially compared to the Wiener model (a difference of 0.8446°). For NRMSE, CB-TCN has a value of 0.0932, which is lower than the other models.

(4) Flexion–Extension Motion

Knee Joint: The R^2^ of the CB-TCN model is 0.9717, which outperforms the other models, particularly the Wiener model, with a difference of 0.1042. Compared to the second-best model, ED-TCN (R^2^ = 0.9579), CB-TCN also demonstrates superior performance. The RMSE is 1.5891°, significantly lower than the other models, especially the Wiener model (a difference of 2.2909°). For NRMSE, CB-TCN has a value of 0.05583, which is lower than the other models.

Ankle Joint: The R^2^ of the CB-TCN model is 0.9459, significantly higher than the other models, especially the Wiener model, with a difference of 0.1042. Compared to the second-best model, ED-TCN (R^2^ = 0.9114), CB-TCN also shows a notable performance advantage. The RMSE is 5.1833°, lower than the other models, particularly the Wiener model (a difference of 6.0915°). For NRMSE, CB-TCN has a value of 0.07585, which is lower than the other models.

The CB-TCN model outperforms all other models across all motion types, particularly in terms of R^2^, RMSE, and NRMSE, demonstrating higher prediction accuracy and smaller errors. The combined analysis of *p*-values for R^2^ and RMSE, along with the Tukey HSD test, reveals that the proposed CB-TCN model exhibits significant differences in both fitting ability and predictive performance compared to other models. In gait motion, CB-TCN shows particularly excellent performance in the hip, knee, and ankle joints, with high precision and small errors. These joints play a crucial role in normal walking, where hip flexion–extension and knee flexion provide primary motion, while the ankle joint plays a critical role in propulsion and shock absorption. The model’s high accuracy reflects its ability to accurately capture the coordinated motion of these joints in routine, low-dynamic movements.

However, in more complex movements such as obstacle crossing, squatting, and flexion–extension motions, the model exhibits some deviations in prediction accuracy. These deviations are primarily due to the varying dynamic range and non-linear muscle activation patterns during these movements, leading to slightly higher RMSE and NRMSE values. From a physiological perspective, obstacle crossing and squatting involve more complex muscle coordination, particularly the rapid changes in body weight distribution and stability, requiring higher degrees of muscle activation and joint coordination. Specifically, during squatting, the rectus femoris (RF) and biceps femoris (BF) must work in high synchronization to control knee flexion and maintain a stable posture. The rapid fluctuations in joint angles, particularly during deep squats, challenge the model’s ability to capture these dynamic changes. Additionally, the gastrocnemius (GAS) and soleus (SOL) muscles work together to stabilize the ankle joint during squatting and obstacle crossing. Due to changes in load and posture, the activation patterns of these muscles are highly non-linear, further increasing the prediction difficulty. In flexion–extension movements, the model also faces challenges because muscle activation patterns are typically non-linear and vary significantly with changes in movement speed, load, and posture. Muscles like the tibialis anterior (TA), rectus femoris (RF), and biceps femoris (BF) undergo rapid changes during flexion–extension, which significantly increases the complexity of the model’s prediction capability, especially under rapidly changing joint angles and load conditions.

Despite these challenges, the CB-TCN model still demonstrates overall lower RMSE and NRMSE compared to other models, with R^2^ remaining high, indicating strong generalizability.

### 4.3. Real-Time Analysis

Table 1 presents the computation time (in milliseconds) required per operation for the model proposed in this study across different joints (hip, knee, ankle) and movements (walking, obstacle crossing, squatting, knee flexion–extension). The data are presented as mean ± standard deviation, reflecting the average inference time and stability of the model across multiple experiments.

As shown in the table above, the inference time of the model proposed in this study is consistently below 50 ms under all test conditions, meeting the real-time requirements. In the walking motion, the model exhibits the lowest inference time, demonstrating its excellent performance in simple motion tasks. In more complex motions, such as obstacle crossing, the model still maintains a high level of efficiency. These results indicate that the model can adapt to various motion modes and meet the demands for real-time feedback. All networks in this study were implemented using Python 3.8 and PyTorch 2.4.1. The training and evaluation processes were conducted on a computer equipped with an NVIDIA GeForce RTX 4060 GPU, ensuring efficient computation and reliable performance.

## 5. Conclusions

In this study, the focus was on predicting joint angles in lower-limb movements using the CB-TCM model, applying it to rehabilitation and motion analysis. Predicting joint angles is essential for advancing rehabilitation technologies, particularly for individuals with impaired motor function due to injury or neurological conditions. By accurately modeling joint movements, this approach facilitates the development of adaptive therapies, improves the design of assistive devices like exoskeletons and prosthetics, and enhances the understanding of human biomechanics. The method involves training the model with data from walking, obstacle crossing, squatting, and knee flexion–extension movements, and evaluating it using key metrics such as R^2^, RMSE, and NRMSE. The model performs excellently across all movements, particularly in walking and knee extension. During walking, the knee joint achieved an R^2^ value of 0.9718, with an RMSE as low as 1.2648°. The model still performs well in squatting and obstacle crossing tasks, despite slight increases in RMSE and NRMSE.

In future research, we aim to evaluate the practical feasibility of the proposed model in clinical environments, focusing on its applicability to different patient populations and ensuring user-friendliness. To enhance the generalizability of our findings, we plan to expand the participant pool to include individuals with varying health conditions, ages, genders, and body types. This will help verify the model’s broad applicability and explore its performance across different populations, ensuring its sustainability, safety, and usability in real-world applications. Additionally, we will assess the model’s performance across different clinical settings and patient groups, such as those with neurological impairments, movement disorders, or limb deficiencies, to explore its potential challenges and benefits. To ensure its clinical viability, we will address several critical aspects, including the compatibility of the technology with existing medical equipment and rehabilitation tools, the impact of various factors (such as skin condition and sensor placement) on signal quality, and the development of a user-friendly interface for both healthcare professionals and patients. Furthermore, safeguarding patient data privacy and ensuring secure data handling will be a priority to comply with ethical and regulatory standards.

## Figures and Tables

**Table 1:** Prediction time for joint movements under different types of exercises (unit: ms).

**Activity**	**Hip Joint (ms)**	**Knee Joint (ms)**	**Ankle Joint (ms)**
Walking	13.20 ± 0.048	13.53 ± 0.124	13.46 ± 0.056
Obstacle Crossing	34.59 ± 0.068	34.31 ± 0.044	34.01 ± 0.072
Squatting	28.68 ± 0.064	28.68 ± 0.076	27.77 ± 0.060
Knee Flexion-Extension	27.17 ± 0.060	27.18 ± 0.048	27.18 ± 0.048

## Data Availability

If data are needed, please contact the corresponding author.

## Supplementary Materials

The following supporting information can be downloaded at: https://www.mdpi.com/article/10.3390/s25030719/s1, Table S1: Model structure parameters; Table S2: Statistical indicators.
